# Acylated Ghrelin Renders Chemosensitive Ovarian Cancer Cells Resistant to Cisplatin Chemotherapy via Activation of the PI3K/Akt/mTOR Survival Pathway

**DOI:** 10.1155/2019/9627810

**Published:** 2019-07-08

**Authors:** Attalla Farag El-kott, Ali A. Shati, Mohammed Ali Al-kahtani, Sultan Alqahtani

**Affiliations:** ^1^Biology Department, College of Science, King Khalid University, Abha, Saudi Arabia; ^2^Zoology Department, College of Science, Damanhour University, Egypt; ^3^Department of Basic Medical Sciences, King Saud bin Abdulaziz University for Health Sciences, Riyadh, Saudi Arabia; ^4^King Abdullah International Medical Research Center, Riyadh, Saudi Arabia

## Abstract

This study investigated the effect of acylated synthetic ghrelin (AG) on the survival and proliferation of human chemosensitive ovarian cancer cells (A2780) and explored some mechanisms of action with a focus on the p53 apoptotic pathway and PI3K/Akt and NF-*κ*B survival pathways. Human A2780 ovarian cancer cells were cultured with or without AG treatment in the presence or absence of cisplatin. In some cases, cisplatin+AG-treated cells were pre-incubated either with [D-Lys3]-GHRP-6, a ghrelin receptor antagonist, or with LY294002, a PI3K inhibitor. mRNA of ghrelin receptors(GHS-R1a and GHS-R1b), as well as, protein levels of GHS-R1a, were expressed abundantly in A2780 cells. AG treatment did not affect the mRNA and protein levels of GHS-R1a and GHS-R1b in both control and Cis-treated cells. However, while AG treatment had no effect on control cell viability, it significantly increased cell viability and proliferation and inhibited cell death in Cis-treated cells. In both control and Cis-treated cells, AG treatment significantly increased PI3K/Akt/mTOR signaling and enhanced the nuclear accumulation of NF-*κ*B. Concomitantly, in both control and Cis-treated cells, AG significantly lowered the protein levels of p53, p-p53 (Ser16), PUMA, cytochrome C, and cleaved caspase-3. Interestingly, pre-incubating the cells with either [D-Lys3]-GHRP-6 or LY294002 completely abolished the above-mentioned effect of AG in both control and Cis-treated cells. In conclusion, the findings of this study show that AG promotes cell survival of the OC cells and renders them resistat to Cis therapy, an effect that is mediated by the activation of PI3K/Akt/mTOR and activation of NF-*κ*B, and requires GHS-R1a.

## 1. Introduction

Ovarian cancer (OC) is the second most common type of lethal gynecologic malignancies of the female population all throughout the world [1, 2]. In spite of the big improvement in the clinical field for OC therapy, an overlall low survival and high recurrence rates still exist and were attributed to the poor screening approach, inadequate therapeutic options, and development of drug resistance [3, 4]. This suggests that further research is required to understand the molecular mechanisms behind OC development and resistance to achieve a more specific therapeutic approach.

Thanks to the continuous research to understand the molecular aspects of OC in an attempt to better understand and consequently treat the disease Currently, the extensive genomic analysis has identified some common hyperactive intracellular signaling pathways responsible for the development and progression of OC in patients [5, 6]. Among them and over decades of research, it was shown that the PI3K/Akt/mTOR pathway is one of the most frequently hyperactive pathways in OC as well as other types of solid tumors and is associated with increased cell growth, proliferation, migration, protein synthesis, survival, and chemotherapy resistance [7–9]. On the other hand, particular attention has been paid recently to the role of gut hormones in the tumorigenesis of various types of solid tumors. Among a ll, accumulating evidence has shown that ghrelin, an endogenous ligand for growth hormone secretagogue receptor (GHSR), may play a crucial role in the development and progression of numerous kinds of solid tumors [10]. Ghrelin is mainly secreted from stomach cell and circulates in the blood in two forms. Acylated ghrelin (AG, n-octanoylated at Ser3) is the most active but less abundant form of circulatory ghrelin and was reported to act peripherally by activating secretagogue receptor type 1a (GHS-R1a). On the other hand, the unacylated ghrelin (UAG) which is the most abundant but less active form of ghrelin which acts independently of GHS-R1a receptors [11]. In mammals, acylation is achieved by the activation of GOAT enzymes in the stomach [11].

Interestingly, mRNA of AG and its own receptor, GHS-R1a, are widely expressed, but to a less extent, in a variety of normal tissues [12]. However, current evidence has shown overexpression of AG and its receptor subtypes, including GHS-R1a or 1b, or both, in a great number of solid tumors, including pituitary, thyroid, breast, lung, and prostate adenomas, gonadal germ cell tumors, and gastric and colorectal cancer [12, 13]. Hence, although some few studies support the antiproliferation and protective action of AG against some types of solid tumors at higher ghrelin doses, the above-mentioned studies, which used a normal serum concentration of ghrelin in vitro, support that AG promotes carcinogenesis by enhancing motility, proliferation, and invasiveness of cancerous cells. In support, pharmacological inhibition of AG effect by D-Lys-GHRP6, a specific GHS-R1a blocker, or by SB801, a selective AG antibody, inhibited and suppressed the proliferation and invasion of cancerous colorectal cell lines induced by AG [14].

mRNA of the AG and GHS-R1a has been detected in the ovaries of both human and rodents a and was also highly expressed in HO-8910 OC cells [15]. The effect of high circulatory or exogenous AG on the tumorigenesis of OC is poorly studied, and findings are largely lacking. In a single study, ghrelin was shown to induce apoptosis and inhibit the growth of OC by the activation of ERK signaling in a GHS-R1a-dependent mechanism [16]. In this study, the authors used a higher serum concentration of AG (121-242 nM) to stimulate the cultured cells. However, the impact of AG on OC cell tumor growth at a concentration similar to serum levels is not investigated yet. Interestingly, at a very low dose (10^−8^-10^−7^ M), AG activated the PI3K/Akt signaling pathway in cardiac microvascular endothelial cells [17]. Also, treatment of colon cancerous cells with low levels of ghrelin (0.1-10 nM) cells stimulated colon cancer cell proliferation via the activation of the Ras/PI3K/Akt/mTOR signaling pathway, an effect that was shown to be completely dependent to GHS-R1a [18].

Based on these facts, in this study, it was in our interest to investigate the effect of low dose of AG (1 nM) on cell death and proliferation of human chemosensitive ovarian cancer cell lines (A2780) in the presence or absence of cisplatin (Cis), a common chemotherapeutic agent, with the emphasis on its effect to PI3K/Akt/mTOR signaling axis.

## 2. Materials and Methods

### 2.1. Drug Preparations

Cisplatin (Cat. No. 15663-27-1), AG (Cat. No. G8903), LY294002 (Cat. No. L9908), and [D-Lys3]-GHRP-6 (Cat. No. G4535) were purchased from Sigma-Aldrich. Ghrelin and [D-Lys3]-GHRP-6 were always dissolved in the 0.9% NaCl and used freshly. LY294002 was initially dissolved in DMSO solution and diluted in the culture media to a concentration used in the study. The final concentration of DMSO in the media was 0.02%.

### 2.2. Cell Culture

Human chemosensitive ovarian cancer cells (A2780) (Cat. No. 93112519) were purchased from Sigma-Aldrich, UK. A2780 cells were cultured at 37°C in a humidified atmosphere (5% CO2) as previously described by Fraser et al. (2006). Accordingly, cells were maintained in Dulbecco's modified Eagle's medium (DMEM/F12) (ThermoFisher Scientific Inc., Rockford, IL, USA) supplemented with 10% fetal bovine serum, 50 g/ml streptomycin, 50 U/ml penicillin, 1% nonessential amino acids, and 0.625 g/ml fungizone. Cells were plated at a density of 5 × 10^4^ cells in 60 mm dishes, 18 h before the beginning of experimental treatments. At the time of treatment, the cell density was <85% confluent.

### 2.3. Cell Treatments

At the time of the experimental procedure, the culture media were changed with new ones and treated as follows: (1) control: incubated in the culture media for 72 hours in the presence of the vehicle (normal saline), (2) AG-treated: initially, pre-incubated with AG (1 nM) for 24 hours and then incubated for the next 72 hours with new media containing the vehicle, (3) Cis-treated cells: incubated with Cis (10 *μ*M) for 72 hours, and (4) AG+Cis-treated cells: pre-incubated with AG for 24 hours and then incubated for the next 72 hours with new media containing 10 *μ*M Cis. To investigate if the effect of AG is mediated through GHS-R1a and involves the activation of the PI3K/Akt signaling pathway, another set of cells was pretreated for 1 hour with a medium containing 10 *μ*M of ghrelin receptor antagonist ([D-Lys3]-GHRP-6) or 20 *μ*M of the PI3K inhibitor, LY294002, before being treated as in groups 3 and 4. The doses of AG , [D-Lys3]-GHRP-6, and LY294002 were adopted from the study of Lien et al. [18]. The dose of Cis which was used in this study was shown previously by Fraser et al. [19] who showed it to induce apoptosis in A2780. The dose of [D-Lys3]-GHRP-6 and LY294002 was based on the study of Lien et al. [18].

### 2.4. Cell Viability Assay

The cell viability in all treated cultured cells was performed using the MTT reagent (tetrazolium blue thiazol-3-[4,5-dimethyl-thiazol-2-yl]-2,5-diphenyl-tetrazolium) assay. MTT reagent (20 *μ*l of 5 mg/ml) was added to each well, and the cells were incubated for an extra 4 hours after. The media was then removed, and 100 *μ*l of DMSO was added to each well for 10 minutes to dissolve formazan crystals. The absorbance was measured at 570 nm. All procedures were done in triplicates for 6 samples/treatment.

### 2.5. BrdU Cell Proliferation Analysis

BrdU cell proliferation analysis was done on all experimental groups using the 5-bromo-2′-deoxyuridine (BrdU) colorimetric kit (Cat. No. 11647229001; Roche Diagnostics, Indianapolis, IN, USA). The principle of the test relies on the ability of BrdU to incorporate into newly synthesized cellular DNA and the ability of anti-BrdU-peroxidase (POD) to bind to it, in which the whole immunocomplex can be detected using the 3,3′, 5,5′-tetramethylbenzidine substrate. Briefly, at the end of all incubation, 10 *μ*M BrdU was added to each well and was incubated at 37°C for an extra 2 hours. Then, an anti-BrdU-peroxidase (POD) working solution was added to each well and incubated at room temperature for another 90 minutes. The resultant absorbance of each reaction was measured using an ELISA reader at 370. Absorbance at 492 nm was used as a reference range. All experiments were done in duplicate for 6 samples/treatment and according to the manufacturer's instruction and presented as % of control.

### 2.6. Quantitative Measurement of Apoptosis by ELISA

Quantitative assessment of apoptosis was performed using a Cell Death Determination ELISA Kit (Cat. No. 11544675001, Roche Diagnostics GmbH, Mannheim, Germany). The kit measures the cytoplasmic histone-associated DNA fragments (mono- and oligonucleosomes) in cytoplasmic fractions. The test is based on the ability of the antihistone antibody to react with the histones H1, H2A, H2B, H3, and H4 and the concurrent ability of peroxidase-conjugated DNA antibody (anti-DNA POD) to bind single- and double-stranded DNA. The kit utilizes coating, incubating, conjugation, and washing buffers as well as the substrate solution. At the end of procedures, the developed absorbance of each reaction was read using an ELISA reader at 405 nm. Absorbance at 490 nm was used as a reference range. All procedures were done in duplicate of 6 samples/treatment, according to the manufacturer's instructions.

### 2.7. Real-Time Polymerase Chain Reaction

The primer sequence used for measuring the mRNA of GHS-R1a and GHS-R1b was adopted from the study according to Cassoni et al. [20]. The forward primer for both genes was 5′-TCGTGGGTGCCTCGCT-3′ whereas the reverse primers for GHS-R1a and GHS-R1b were 5′-CACCACTACAGCCAGCATTTTC-3′ and GCTGAGACCCACCCAGCA-3′, respectively. The expected sizes of the amplicons of GHS-R1a and GHS-R1b were 65 bp and 66 bp, respectively. Total RNA was extracted from frozen platelets using an RNeasy Mini Kit (Cat. No. 74104, Qiagen, Victoria, Australia). The purities and concentrations of the RNA in all samples were determined using a NanoDrop spectrophotometer (ThermoFisher, MA, USA). A single-stranded cDNA was synthesized from all samples using a Superscript II reverse transcriptase kit with oligo (dT) primers (Cat. No. 18064014, ThermoFisher, MA, USA). Ssofast Evagreen Supermix (Cat. No. 172-5200, Bio-Rad, Montreal, Canada) was used to monitor the amplification using a CFX96 real-time PCR system (Bio-Rad, CA, USA). All procedures were carried out in accordance with the manufacturer's instructions. In every plate, the template DNA was omitted as a positive control. The relative mRNA level of each gene was relatively expressed to its corresponding *β*-actin mRNA level.

### 2.8. Western Blotting Procedure

Total proteins in total cell homogenates were extracted from cell groups using M-PE mammalian protein extraction reagent (Cat. No. 78501, ThermoFisher Scientific Inc., Rockford, IL, USA). The NE-PER Nuclear and Cytoplasmic Extraction kit (Cat. No. 78835, ThermoFisher Scientific) was used to prepare both fractions from cultured cells. Levels of cytochrome C were measured in the cytoplasmic fraction. Levels of NF-*κ*B P65 were determined in both the cytoplasmic and nuclear fractions. Protein levels in all fractions/samples were detected using a Pierce BCA Protein Assay Kit (Cat. No. 23225, ThermoFisher Scientific Inc., Rockford, IL, USA). Equal amounts of proteins (40 *μ*g/well) were loaded and resolved by 81-12% SDS-PAGE and electrotransferred (100 V, 2 hours) onto nitrocellulose membranes (Bio-Rad). Membranes were then blocked with skim milk (5%; *w*/*v*, prepared in 0.05% Tween 20 buffer (TBST)) for 1 hour at room temperature. Membranes were then blotted with primary antibodies (Table 1) for 2 hours at room temperature on a shaker. Membranes were then washed with TBST buffer and incubated for another 2 hours at room temperature with the corresponding HRP-conjugated secondary antibodies. Antigen-antibody interactions were detected using an enhanced chemiluminescence detection kit (Pierce ECL reagents, ThermoFisher Scientific Inc., Rockford, IL, USA) and then scanned and analyzed using the C-DiGit blot scanner (LI-COR, NE, USA) and associated software. Membranes were stripped up to 4 times, and the detection of the phosphorylated forms was done first. Individual band densities were adjusted between different blots for intergel variability using an internal standard. Protein levels of phosphorylated and total proteins were presented relative to that of *β*-actin. Activation ratios of desired proteins were calculated as the relative expression of phosphorylated protein divided by total protein levels.

### 2.9. Statistical Analysis

Statistical analysis for all measured parameters was done using the GraphPad Prism statistical software package (version 6). Differences among the experimental groups were assessed by one-way ANOVA, followed by Tukey's test. Data were presented as mean ± SD. Values will be considered significantly different when *P* < 0.05.

## 3. Results

### 3.1. AG Promotes Cell Proliferation and Survival and Inhibits Cell Apoptosis in Cis-Treated A2780 OC Cells through GHS-R1a but without Affecting the Expression of GHS-R1a

**Figure 1:**
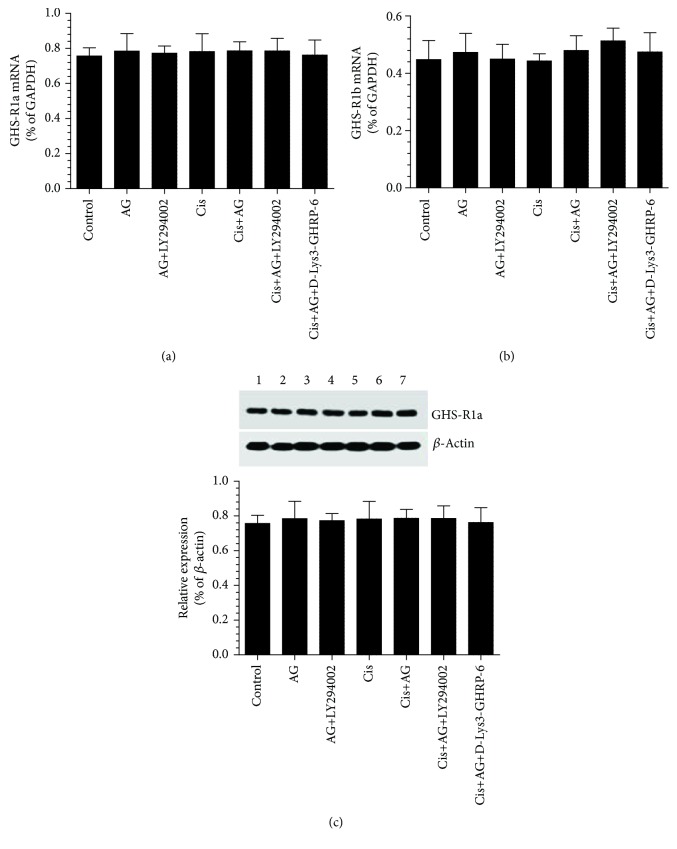
Changes in mRNA of ghrelin receptor GHS-R1a and GHS-R1b as well as in protein levels of GHS-R1a receptors in control and cisplatin- (Cis-) treated cultured human chemosensitive ovarian cancer cells (A2780). Cells were grown in DMEM/F12 media containing AG ghrelin (1 nM) for 24 hours, then transferred to a medium containing the vehicle for the next 72 hours. For Cis treatment, cells were grown in the same medium containing Cis (10 *μ*M) for 72 hours. In addition, AG and Cis-treated cells were also preincubated with 10 *μ*M ghrelin receptor antagonist, [D-Lys3]-GHRP-6, and/or 20 *μ*M of the PI3K inhibitor, LY294002. Control cells were grown in the presence equivalent volume of normal saline. Results are shown as the mean ± SD of *n* = 6 experiments. ^a^: vs control (lane 1), ^b^: vs AG (lane 2), ^c^: vs AG+LY294002 (lane 3), ^d^: vs Cis (lane 4), ^e^: vs AG+Cis (lane 5). Lane 6: Cis+AG+LY294002 and lane 7: Cis+AG+[D-Lys3]-GHRP-6.

**Figure 2:**
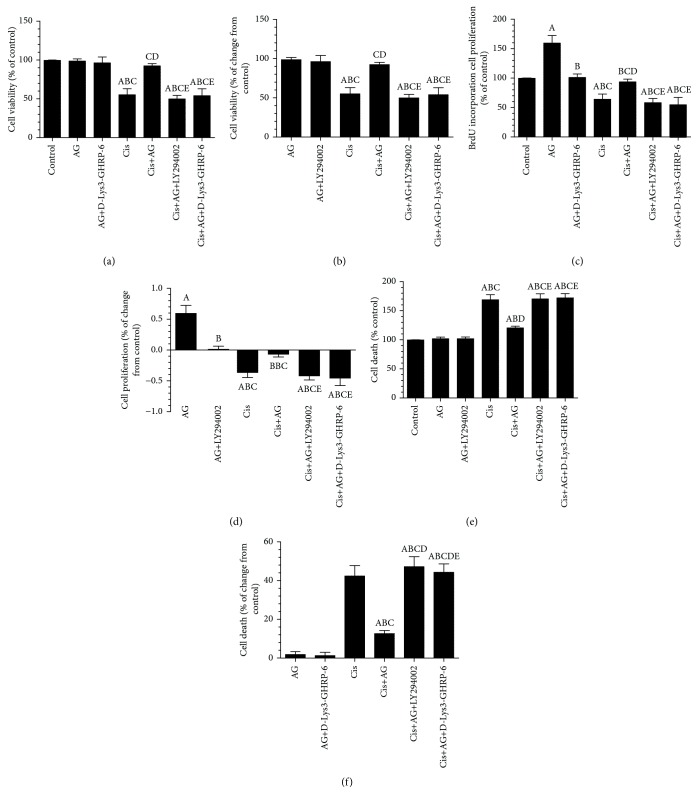
Acylated ghrelin (AG) induces cell proliferation and prevents cisplatin- (Cis-) induced cell death in cultured human chemosensitive ovarian cancer cells (A2780). Cells were grown in DMEM/F12 media containing AG ghrelin (1 nM) for 24 hours, then transferred to a medium containing the vehicle for the next 72 hours. For Cis treatment, cells were grown in the same medium containing Cis (10 *μ*M) for 72 hours. In addition, AG and Cis-treated cells were also preincubated with 10 *μ*M ghrelin receptor antagonist, [D-Lys3]-GHRP-6, and/or 20 *μ*M of the PI3K inhibitor, LY294002. Control cells were grown in the presence equivalent volume of normal saline. Results are shown as the mean ± SD of *n* = 6 experiments. ^a^: vs control, ^b^: vs AG, ^c^: vs AG+LY294002, ^d^: vs Cis, ^e^: vs AG+Cis.

As shown in Figures 1(a)–1(c), the mRNA levels of GSH-R1a and GHS-R1b, as well as the protein levels of GHS-R1b, were not significantly different between all groups of treatment, thus suggesting that the inhibitory effect of Cis or the stimulatory effect of AG on cell survival do not involve regulation of the transcription or translation of GHS-R1a/1b. However, the cell survival and proliferation were significantly decreased, whereas apoptosis rate was significantly increased in Cis-treated cells, as compared to control cells. However, there were no significant change in survival rate or apoptosis ratio, but cell proliferation was significantly increased in control+AG-treated cells as compared to control cells which received the vehicle (Figures 2(a)–2(f)). On the other hand, control+AG+D-Lys3]-GHRP-6 showed no alteration in cell survival or apoptosis ratio but had significantly lowered cell proliferation ratio, as compared to control+AG-treated cells (Figures 2(a)–2(f)). Furthermore, Cis+AG-treated cells showed a significant increase in cell survival and proliferation ratios and a significant decrease in cell death ratio, as compared with Cis-treated cells (Figures 2(a)–2(f)). These data suggest that AG is able to inhibit Cis-induced cell death. However, there were a significant increase in cell death ratio and a significant decrease in cell survival and proliferation ratios in both AG+Cis+LY294002 and AG+Cis+[D-Lys3]-GHRP-6, as compared to AG+Cis-treated cells. Interestingly, the ratio of cell proliferation, apoptosis, and survival in AG+Cis+LY294002 or AG+Cis+[D-Lys3]-GHRP-6 was not significantly different as compared to each other or when compared to Cis-treated cells. These data suggest that the stimulatory effect of AG does not require modulating the expression of GHS-R1a but needs the presence of GHS-R1a and is mediated by the activation of PI3K.

### 3.2. AG Enhances the Levels and Activity of PI3K/Akt in Control and Cis-Treated A2780 OC Cells through GHS-R1a

**Figure 3:**
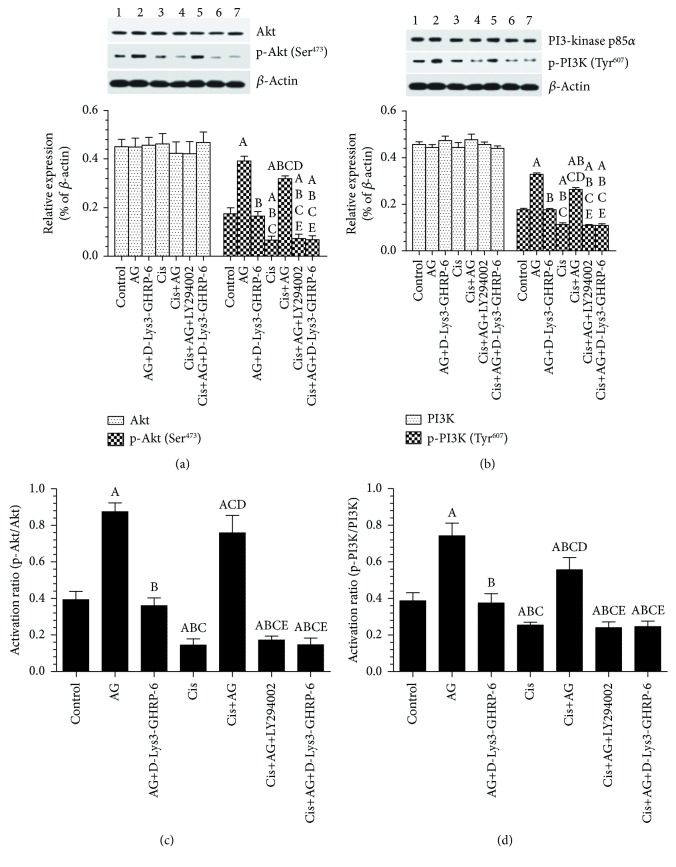
Acylated ghrelin (AG) activates the PI3K/Akt signaling pathway in control and cisplatin- (Cis-) treated cultured human chemosensitive ovarian cancer cells (A2780). Cells were grown in DMEM/F12 media containing AG ghrelin (1 nM) for 24 hours, then transferred to a medium containing the vehicle for the next 72 hours. For Cis treatment, cells were grown in the same medium containing Cis (10 *μ*M) for 72 hours. In addition, AG and Cis-treated cells were also preincubated with 10 *μ*M ghrelin receptor antagonist, [D-Lys3]-GHRP-6, and/or 20 *μ*M of the PI3K inhibitor, LY294002. Control cells were grown in the presence equivalent volume of normal saline. Results are shown as the mean ± SD of *n* = 6 experiments. ^a^: vs control (lane 1), ^b^: vs AG (lane 2), ^c^: vs AG+LY294002 (lane 3), ^d^: vs Cis (lane 4), ^e^: vs AG+Cis (lane 5). Lane 6: Cis+AG+LY294002 and lane 7: Cis+AG+[D-Lys3]-GHRP-6.

**Figure 4:**
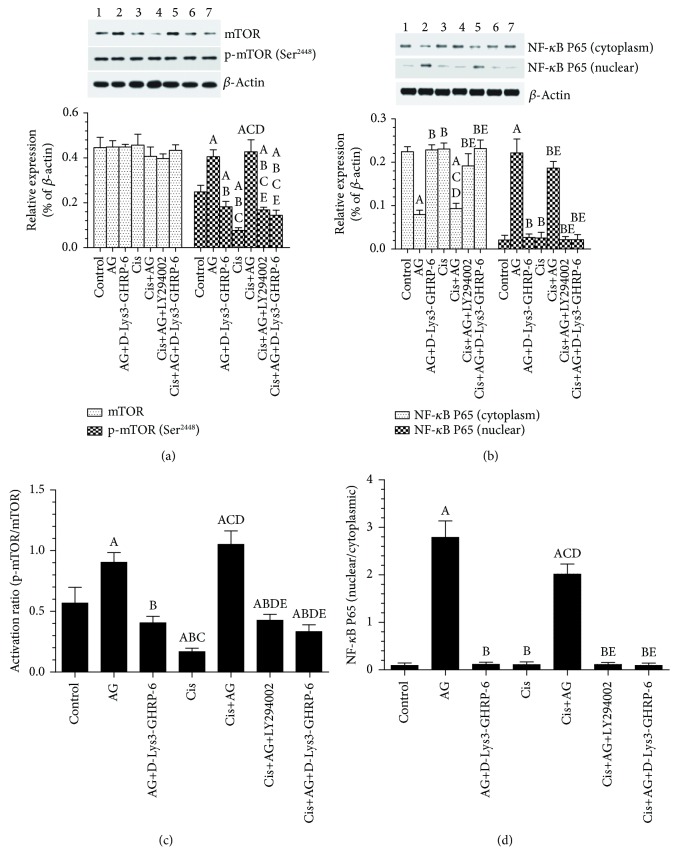
Acylated ghrelin (AG) activates the mTOR signaling pathway and stimulates NF-*κ*B nuclear translocation in control and cisplatin- (Cis-) treated cultured human chemosensitive ovarian cancer cells (A2780). Cells were grown in DMEM/F12 media containing AG ghrelin (1 nM) for 24 hours, then transferred to a medium containing the vehicle for the next 72 hours. For Cis treatment, cells were grown in the same medium containing Cis (10 *μ*M) for 72 hours. In addition, AG and Cis-treated cells were also preincubated with 10 *μ*M ghrelin receptor antagonist, [D-Lys3]-GHRP-6, and/or 20 *μ*M of the PI3K inhibitor, LY294002. Control cells were grown in the presence equivalent volume of normal saline. Results are shown as the mean ± SD of *n* = 6 experiments. ^a^: vs control (lane 1), ^b^: vs AG (lane 2), ^c^: vs AG + LY294002 (lane 3), ^d^: vs Cis (lane 4), ^e^: vs AG+Cis (lane 5). Lane 6: Cis+AG+LY294002 and lane 7: Cis+AG+[D-Lys3]-GHRP-6.

Stable total protein levels of PI3K and Akt (Figures 3(a) and 3(b)), as well as in total protein levels of mTOR, were detected in cells of all treatments (Figures 3(a)–3(d) and Figures 4(a) and 4(c)). On the other hand, there were a significant decrease in the protein levels of p-PI3K (Tyr^607^), p-Akt (Ser^473^), p-mTOR (ser^2448^), and their activation ratios in Cis-treated cells, as compared to control cells (Figures 3(a)–3(d) and Figures 4(a) and 4(c)). These data suggest that Cis-induced cell death is mediated by inhibiting the activity of PI3K/Akt/mTOR. However, control+AG or Cis+AG-treated cells showed a significant increase in the protein levels of p-PI3K (Tyr^607^), p-Akt (Ser^473^), and p-mTOR (ser^2448^), as well as in their activation ratios as compared to control or Cis-treated cells, respectively (Figures 3(a)–3(d) and Figures 4(a) and 4(c)). These data suggest that AG mainly act by increasing the activity of PI3K/Akt/mTOR signaling. Of interest, control+AG+[D-Lys3]-GHRP-6 had significantly lower levels of p-PI3K (Tyr^607^), p-Akt (Ser^473^), p-mTOR (ser^2448^), and their activation ratios as compared to control+AG-treated cells (Figures 3(a)–3(d) and Figures 4(a) and 4(c)). Similarly, Cis+AG+[D-Lys3]-GHRP-6 or Cis+AG+LY294002-treated cells had significantly lower levels of p-PI3K (Tyr ^607^), p-Akt (Ser^473^), p-mTOR (ser^2448^), and their activation ratios as compared to Cis+AG-treated cells (Figures 3(a)–3(d) and Figures 4(a) and 4(c)). These data suggest that AG-stimulated PI3K/Akt/mTOR is essential for cell survival and such effect requires the presence of GHS-R1a receptors.

### 3.3. AG Stimulates the Nuclear Accumulation of NF-*κ*B in Control and Cis-Treated A2780 OC Cells through GHS-R1a

Abundant cytoplasmic levels and low nuclear/cytoplasmic ratio of NF-*κ*B were seen in control cells. Cis treatment did not affect the cytoplasmic and nuclear distribution of NF-*κ*B P65, as compared to control cells (Figures 4(b) and 4(d)). Control+AG or Cis+AG-treated cells showed higher nuclear levels and nuclear/cytoplasm ratio of NF-*κ*B P65, as compared to control or Cis-treated cells, respectively (Figures 4(b) and 4(d)). However, control+AG+[D-Lys3]-GHRP-6-treated cells had a significant increase in the cytoplasmic levels and a significant decrease in the nuclear levels of NF-*κ*B P65 as compared to control+AG-treated cells. A similar significant decrease in the nuclear accumulation of NF-*κ*B P65 with a concomitant significant increase in the cytoplasmic levels of NF-*κ*B P65 was seen in Cis+AG+[D-Lys3]-GHRP-6 or Cis+AG+LY294002, as compared to Cis+AG. Of note, there were no significant variations in the cytoplasmic or nuclear levels of NF-*κ*B P65 when all these latter groups were compared to each other (Figures 4(b) and 4(d)). These data suggest that AG, through GHS-R1a, enhances the nuclear accumulation of NF-*κ*B P65 in a mechanism that requires the activation of PI3K.

### 3.4. AG Inhibits the Levels and Activity of p53 and Lowered the Cytoplasmic Levels of PUMA, Cytochrome C, and Cleaved Caspase-3

**Figure 5:**
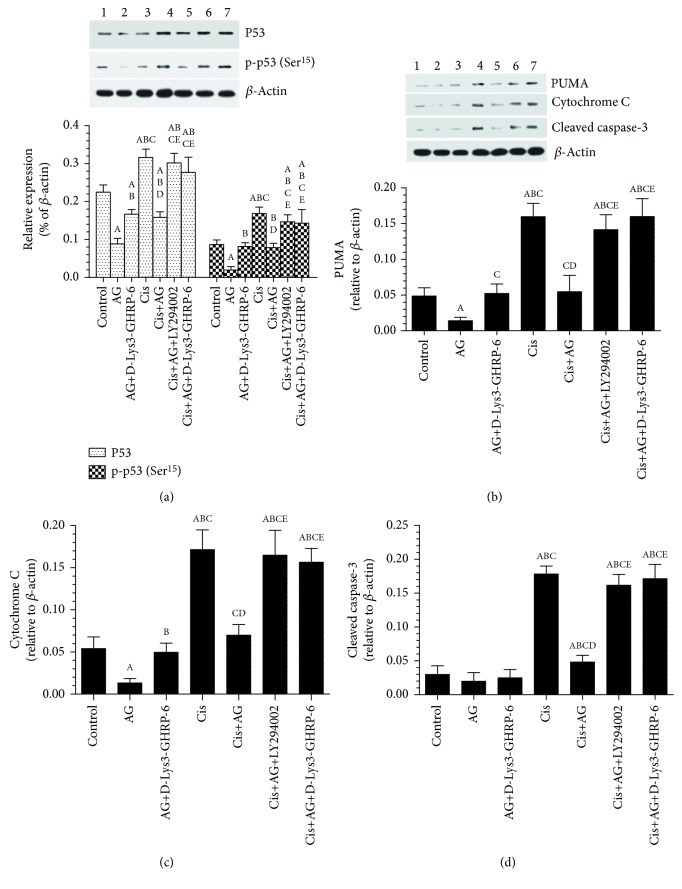
Acylated ghrelin (AG) inhibits p53 levels and activity, cytochrome C release, and activation of cleaved caspase-3 and downregulates PUMA in control and cisplatin- (Cis-) treated cultured human chemosensitive ovarian cancer cells (A2780). Cells were grown in DMEM/F12 media containing AG ghrelin (1 nM) for 24 hours, then transferred to a medium containing the vehicle for the next 72 hours. For Cis treatment, cells were grown in the same medium containing Cis (10 *μ*M) for 72 hours. In addition, AG and Cis-treated cells were also preincubated with 10 *μ*M ghrelin receptor antagonist, [D-Lys3]-GHRP-6, and/or 20 *μ*M of the PI3K inhibitor, LY294002. Control cells were grown in the presence equivalent volume of normal saline. Results are shown as the mean ± SD of *n* = 6 experiments. ^a^: vs control (lane 1), ^b^: vs AG (lane 2), ^c^: vs AG+LY294002 (lane 3), ^d^: vs Cis (lane 4), ^e^: vs AG+Cis (lane 5). Lane 6: Cis+AG+LY294002 and lane 7: Cis+AG+[D-Lys3]-GHRP.

As shown in Figures 5(a)–5(d), Cis-treated cells had a significant increase in the protein levels of p53, p-p53 (Ser^15^), PUMA, cytochrome C, and cleaved caspase-3, as compared to control cells. On the other hand, control+AG or Cis+AG-treated cells had significantly lowered the levels of all these apoptotic markers as compared to control or Cis-treated cells, respectively, thus suggesting that AG is able to inhibit apoptosis in both control and Cis-treated cells (Figures 5(a)–5(d)). However, control+AG+[D-Lys3]-GHRP-6 had significantly higher levels of p53, p-p53 (Ser^15^), PUMA, cytochrome C, and cleaved caspase-3, as compared to control+AG-treated cells. Similar results were also observed in Cis+AG+[D-Lys3]-GHRP-6 and Cis+AG+LY294002, as compared to Cis+AG-treated cells (Figures 5(a)–5(d)). These data suggest th0061t AG inhibition of apoptosis is AG dependent and mediated through the activation of PI3K.

## 4. Discussion

The results of the present study indicate that AG, at low levels (1 nM), stimulates the proliferation of human chemosensitive OC cells (A2780) and rendered them resistance to Cis chemotherapy. This effect is mediated through GHS-R1a and involves, at least, activation of the PI3K/Akt signal transduction pathway and its downstream targets, mTOR and NF-*κ*B, as well as downregulation and inhibition of p53 and p53-upregulated modulator of apoptosis (PUMA).

Unlike other types of solid tumors, the effects of AG on OC cells are largely unknown in both *in vivo* and *in vitro* setting, and limited studies to discuss this issue are currently available. In fact, only one single study has shown that AG, at higher levels than those observed in the serum (121-242 nM), is able to induce apoptosis in HO-8910 OC cells through increasing the activity of ERK1/2, in a mechanism that is dependent on GHS-R1a. On the contrary, the first interesting observation in this study was the stimulatory action of AG at a lower dose (1 nM) on the proliferation and antiapoptotic effects of A2780 chemosensitive OC cells treated or untreated with Cis. In fact, we have found that A2780 OC cells, pre-treated with AG, are resistant to Cis-induced apoptosis. Interestingly, AG alone did not change the rate of death or cell survival in control A2780 OC cells. However, all these effects were mediated through GHS-R1a. Indeed, pre-treating the A2780 OC cells with a preestablished dose of [D-Lys3]-GHRP-6 prevented the proliferative effect of AG in untreated cells and increased cell death in Cis-treated cells. Such variation between our findings and the study of Bai et al. [16] could be related to the difference in cell type and the dose of AG used. It has been previously suggested that AG at such high dose induces cell death due to possible cytotoxicity [10].

Supporting our findings, other authors have presented reliable and convincing evidence to show that AG promotes the proliferation and invasion of cancer cell lines [10]. Indeed, AG promoted cell proliferation in prostatic carcinoma cells [21]; medullary thyroid carcinoma [22], hepatoma cell [23], colorectal cancer, and antagonism of AG by D-Lys-GHRP6 or AG-specific antibody (SB801) inhibited such effects [24].

We next aimed to examine the mechanism(s) by which AG induces such effects. For this reason, we first targeted the effect of AG therapy on the expression of GHS-R1 subtypes. Based on our findings, the presence of GHS-R1 receptors was crucial for AG to stimulate cell survival in these cancer cells type. However, it does not affect its expression at the transcription or translational levels, leaving the doubts about the regulation of these receptors during OC development, which is out of the scope of this study.

We also targeted to investigate the effect of AG on the PI3K/Akt signal transduction pathway and some of its downstream targets. This was based on the crucial role of this pathway as a key regulator of essential cellular processes, including cell survival, growth, differentiation proliferation, senescence, and angiogenesis in various tissues [25, 26]. In addition, there is a consensus that the PI3K/Akt signaling transduction pathway is hyperactivated in most solid tumors, including OC, and is a major contributor of OC tumorigenesis, progression, and chemotherapy drug resistance [8, 9, 27]. Indeed, pharmacological inhibition of PI3K/Akt or any of their downstream targets enhanced the vulnerability of the cancerous cells to chemotherapy, radiotherapy, and hormonal treatment [10, 26].

In fact, the survival signal stimulated by the PI3K/Akt signaling is mediated by the activation of multiple downstream survival targets, including mTOR, NF-*κ*B, and Bad, and inhibition of the apoptotic signal initiated by p53 [28, 29]. In this regard, it was shown that sustained activation of the NF-*κ*B signaling pathway in solid tumors enhances the expression of numerous regulatory genes that promotes cell division (e.g., cyclin D1 and c-Myc), invasion (e.g., matrix metalloproteinases (MMP), urokinase-type of plasminogen activator (uPA), and interleukin-8 (IL-8)), cell survival (e.g., survivin, Bcl-2, and Bcl-xL), cell growth, and proliferation [28]. On the other hand, activation of mTOR induces cell proliferation, metastasis, angiogenesis, and protein translation and growth phosphorylating other several downstream proteins [30, 31].

Also, it was shown that Cis induces cell apoptosis to chemosensitive cancerous cells via upregulation and activation of p53, and higher levels of functional p53 are associated with more chemosensitivity and improved clinical outcome in OC patients [20, 32, 33]. However, p53-mediated apoptosis is completely dependent on its nuclear accumulation that is stimulated by specific several phosphorylations of its own serine (Ser) residues, including Ser^15^, Ser^20^, and Ser^37^ [34, 35]. Accordingly, apoptosis induced by p53 occurs via a transcriptional-dependent mechanism that upregulates pro-apoptotic genes, such as Bax, a p53-upregulated modulator of apoptosis (PUMA), and NOXA, and inhibits those of antiapoptotic function such as Bcl-2 and survivin and via a transcription-independent mechanism where p53 can directly bind to Bcl-2 at the membranes of the mitochondria. In both cases, p53 activates intrinsic cell death by stimulating the release of cytochrome C [20]. Among all, recent evidence suggests that upregulation of PUMA is a major mechanism by which Cis induces its apoptotic effect in human OC cells [20, 36]. Interestingly, Akt inhibits p35 nuclear accumulation and activity by inhibiting its own Ser phosphorylation and thus by decreasing the p53 content and/or subcellular localization via phosphorylating the murine double minute-2 (MDM2), thus promoting p53 ubiquitin-dependent proteolysis [37–39]. However, the exact mechanism by which Cis induces activation of p53 remains largely unknown.

A2780 OC cells normally present the functional wild type of p53 and are highly sensitive to Cis-induced apoptosis [40]. Similar to these studies and coincided with the increased cell death and lower proliferation rates, levels of p53 and p-p53 (Ser^15^), as well as PUMA and cytoplasmic levels of cytochrome C and cleaved caspase-3, were significantly increased in Cis, treated A2780 OC cells, confirming their sensitivity to Cis-induced apoptosis. This was associated with the significant inhibition of the PI3K/Akt signaling transduction pathway (p-PI3K (Tyr^607^) and p-Akt (Ser^473^)) and its downstream target p-mTOR (Ser^2448^). Hence, in addition to the formation of inter- and intrastrand cross-linked DNA adducts and the subsequent DNA damage, our results may suggest that inhibition of PI3K is an additive mechanism by which Cis induces apoptosis in A2780 OC cells.

On the other hand, AG rendered A2780 OC cells resistant to Cis-induced cell apoptosis by activating the PI3K/Akt signaling pathway and its downstream targets mTOR and NF-*κ*B. This was evident by the increase in the expression levels of p-PI3K (Tyr^607^), p-Akt (Ser^473^), and p-mTOR (Ser^2448^) and the nuclear accumulation of NF-*κ*B P65. Interestingly, AG produced similar effects in A2780 OC control cells, confirming its upregulatory role of this signaling pathway. This could explain why control and Cis-treated with AG has lower levels of p53 and p-p53 (Ser^15^) and increased survival and proliferation rate.

To confirm this effect, pre-incubating the Cis-treated A2780 OC cells with LY294002, 1 hour before being treated with AG, completely abolished the stimulatory effect of AG on the PI3K/Akt/mTOR signaling pathway and concomitantly increased the total and phosphorylated levels of p53 and increased cell apoptosis. Similarly, results were also reported when control or Cis-treated A2780 OC cells were treated with [D-Lys3]-GHRP-6, suggesting that the effect is completely mediated through GHS-R1a. Supporting to our findings, the AG was shown to induce proliferation of HT-29 and Caco-2 colon cancerous cells via activation of Ras/PI3K/Akt/mTOR signaling pathways and was completely dependent on GHS-R1a and independent of GH-IGF axis [18, 41].

Overall, the findings of this study are the first to show that the AG at very low levels can enhance OC cell survivals and render them more resistant to chemotherapy- (Cis-) induced apoptosis. These findings have clinical importance and could explain why some OC patients develop chemotherapy resistance faster than others. Hence, more clinical trials should be conducted, and monitoring AG levels in OC patients should be given more attention during the development and progression of the disease and during chemotherapy treatment.

## Figures and Tables

**Table 1:** Antibodies used in the study.

Antibody	Cat. No.	MW (kDa)	Manufacturer
GHS-R1a	Sc-374515	44, 1 : 1000	Santa Cruz Biotechnology
PI3-kinase p85*α*	sc-1637	85 : 1 : 1000	Santa Cruz Biotechnology
p-PI3K (Tyr^607^)	ab182651	85 : 1 : 500	Abcam
Akt1	sc-5298	62 : 1 : 1000	Santa Cruz Biotechnology
p-Akt (Ser^473^)	9271	60, 1 : 500	Cell Signaling Technology
Cleaved caspase-3	9661	17, 19 : 1 : 250	Cell Signaling Technology
BCl-2	sc-7382	26 : 1 : 1000	Santa Cruz Biotechnology
Bax	sc-7480	23 : 1 : 1000	Santa Cruz Biotechnology
NF-*κ*B P65	sc-8008	65, 1 : 500	Santa Cruz Biotechnology
p53	sc-126	53 : 1 : 500	Santa Cruz Biotechnology
p-p53 (Ser^15^)	9248	53 : 1 : 250	Cell Signaling Technology
mTOR	2972	289 : 1 : 1000	Cell Signaling Technology
p-mTOR (Ser^2448^)	2971	289, 1 : 500	Cell Signaling Technology
PUMA	12450	23, 1 : 250	Cell Signaling Technology
*β*-Actin	4970	45, 1 : 1000	Cell Signaling Technology

## Data Availability

The data used to support the findings of this study are available from the corresponding author upon request.
